# Major pneumothorax during pediatric cardiac MRI procedure under general anesthesia: step-by-step analysis and importance of a well-known environment and material

**DOI:** 10.1186/s12871-023-02375-8

**Published:** 2024-01-02

**Authors:** Quentin Delhez, Laurent Bairy, John Mitchell, Adrien Maseri

**Affiliations:** Department of Anesthesiology, Université Catholique de Louvain, Centre Hospitalier Universitaire UCL Namur site Godinne, Yvoir, Belgium

**Keywords:** Anesthesia, Pediatric, Pneumothorax, Barotraumatism, VILI, Apnea

## Abstract

**Background:**

To perform step-by-step analysis of the different factors (material, anesthesia technique, human, and location) that led to major pneumothorax during an infrequent pediatric cardiac MRI and to prevent its occurrence in the future. Anesthesia equipment used in a remote location is often different than those in operating rooms. For magnetic resonance imaging (MRI), ventilation devices and monitors must be compatible with the magnetic fields. During cardiac MRI numerous apneas are required and, visual contact with the patient is limited for clinical evaluation. Anesthesia-related barotrauma and pneumothorax are rare in children and the first symptoms can be masked.

**Case Presentation:**

A 3-year-old boy with atrial septal defect (ASD) and suspicious partial anomalous pulmonary venous return was anesthetized and intubated to perform a follow up with MRI. Sevoflurane maintenance and ventilation were performed using a circular CO_2_ absorber device, co-axial circuit, and 500 mL pediatric silicone balloon. Apneas were facilitated by Alfentanyl boluses and hyperventilation. A few moderated desaturations occurred during the imaging sequences without hemodynamic changes. At the end of the MRI, facial subcutaneous emphysema was observed by swollen eyelids and crackling snow neck palpation. A complete left pneumothorax was diagnosed by auscultation, sonography examination, and chest radiograph. Pneumo-mediastinum, -pericardium and -peritoneum were present. A chest drain was placed, and the child was extubated and transferred to the pediatric intensive care unit (PICU). Despite the anesthesiologist’s belief that PEEP was minimal, critical analysis revealed that PEEP was maintained at a high level throughout anesthesia. After the initial barotrauma, repeated exposure to high pressure led to the diffusion of air from the pleura to subcutaneous tissues and mediastinal and peritoneal cavities. Equipment check revealed a functional circular circuit; however, the plastic adjustable pressure-limiting valve (APL) closed within the last 30° rotation. The balloon was found to be more rigid and demonstrated significantly reduced compliance.

**Conclusions:**

Anesthetists require proficiency is using equipment in non-OR locations and this equipment must be properly maintained and checked for malfunctions. Controlling the human factor risks by implementing checklists, formations, and alarms allows us to reduce errors. The number of pediatric anesthesia performed routinely appeared to be essential for limiting risks and reporting our mistakes will be a benefit for all who care about patients.

## Introduction/Background 

General anesthesia is a common procedure and numerous equipment have been developed to provide the safer environment possible and to avoid any issues. The equipment used in a remote location is often different than those in operating rooms. It makes the procedure more challenging. For magnetic resonance imaging (MRI), ventilation devices and monitors must be compatible with the magnetic fields. During cardiac MRI, repeated apneas are required and access to the patient for clinical evaluation is limited.

Anesthesia-related barotrauma and pneumothorax are rare in children [1] and the first symptoms can go unnoticed due to mechanical ventilation and high fractional inspired oxygen (FiO_2_). Pneumothorax due to barotraumatism in pediatric is relatively well-documented in the literature. Its incidence is sourced as it occurs mainly in hospitalized patients. Thanks to various studies, we now have a better understanding of respiratory physiology and can therefore avoid aggressive respiratory conditions as much as possible when we taking care of young intubated patients. This article provides a step-by-step analysis and proposes guiding principles that should prevent similar cases from happening.

## Case presentation

**Fig. 1:**
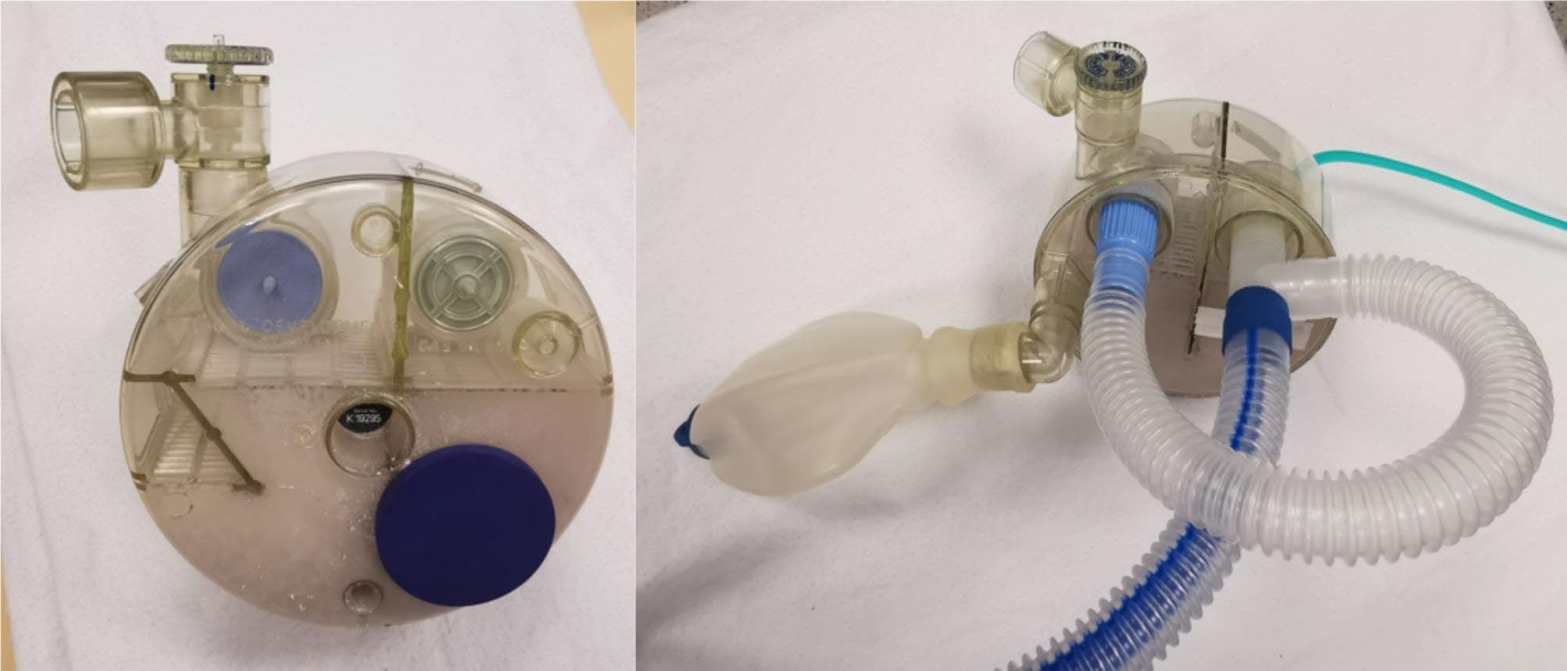
The KAB™ CO_2_ absorber device used to maintain anesthesia during magnetic resonance imaging (MRI). It absorbs CO_2_ in the closed or semi-closed breathing circuit. The green pipe brings fresh gas and permits it to inflate silicon balloons. On the top of the device, there is an APL valve. All are made of plastic (MRI-compatible material)

A 3-year-old child reported to the hospital to undergo a cardiac MRI. The child came for a follow-up of suspicious partial anomalous pulmonary venous return with ASD. He had a few pertinent medical backgrounds. He required follow-up for growth retardation (less than the 3rd percentile) and persistent atrial septal defect. In an outpatient setting, such as for cardiac MRI, after each anesthesia or sedation for the examination, a short monitoring period is scheduled in the PACU. For cardiac MRI, children generally leave the institution at the end of the day. In our institution, anesthesia is induced in a specific room, which contains all the recommended equipment (ventilator, anesthetic equipment, and monitors) to proceed with safe anesthesia. This room is located near the MRI machine (20 m) and allows us to work in a secure environment. The child was induced under general anesthesia using 6% sevoflurane and 100% FiO2. An intravenous catheter was placed after the loss of consciousness. After verifying the good permeability of the peripherical venous catheter, we used propolipid 1% at a dose of 2 mg/kg with alfentanil 10 mcg/kg to proceed with oral intubation. A tracheal cuffed tube with 4.5 mm of internal diameter was used to intubate after spraying 2 mg/kg of lidocaine in the vocal cords. The good position of the tube was confirmed by bilateral symmetric auscultation of the lung and a capnometry monitor. Then, we moved to the MRI room with 4% Sevoflurane gas maintenance and 6 L/min of 50% O_2_/Air. We permanently controlled vital parameters by monitoring SpO_2_, ECG, NIBP, and CO_2_ during the complete examination. Apneas (necessary for cardiac MRI) were induced using short-acting opioids (alfentanil) and by maintaining low end-tidal CO_2_ (EtCO_2_) with hyperventilation. A specific circuit (KAB™ CO_2_ Absorber) (Fig. 1), which was fully MRI compatible, was used.

**Fig. 2:**
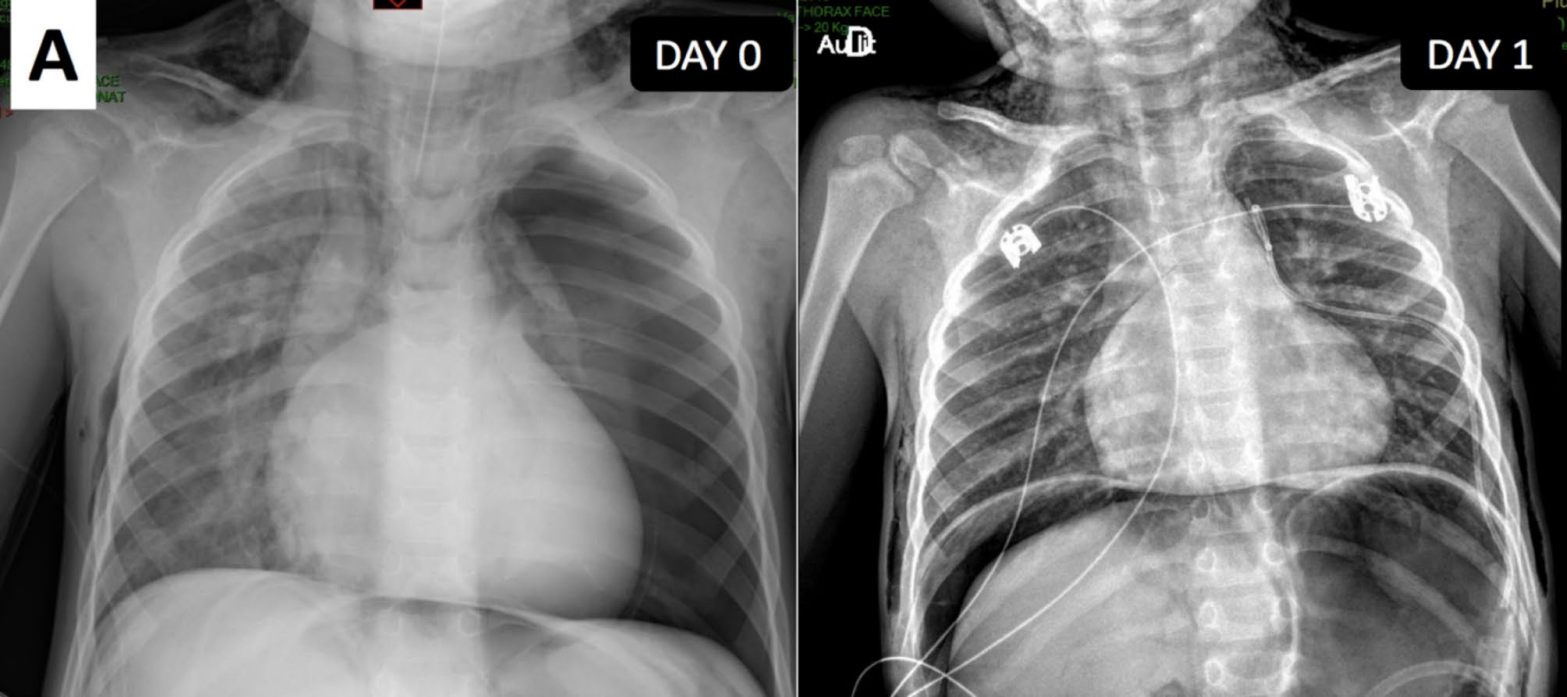
The left thoracic radiograph (Day 0) shows complete left pneumothorax, pneumomediastinum, pneumoperitoneum, and subcutaneous emphysema. On the right radiograph (Day 1), the left lung comes back to its normal position due to the Fuhrman drain. The young patient has been extubated and the major pneumoperitoneum is appreciated

The illustrated circuit is an Ayres/Mapleson D-based circuit with a CO_2_ absorber and a plastic pressure-limiting valve. We used Sevoflurane maintenance for minimizing cardio depressor effects and for limiting the use of propofol in frail patients. Moreover, 25–30 apneas of 10 s each were performed during MRI. Therefore, apneas are followed by periods of rapid manual balloon ventilation to return to physiologic exhaled CO_2_ thresholds. No major incident occurred during the examination such as major desaturation or hemodynamic disturbance (excluding a single episode with 90% of SpO_2_). After the MRI was completed, consistent changes were rapidly observed in our young patient. The cervicofacial region was edematous and oxygenation began to get worse. We observed swollen eyelids and crackling snow in the neck area. After assistance from pediatric anesthetists and assuring adequate hemodynamics, we proceeded with fast sonography to exclude any pneumothorax. The ultrasound machine was readily available. Unfortunately, due to the excessive amount of subcutaneous air, no relevant information could be obtained from this examination. We therefore performed a chest radiograph to remove any doubt, since this is the gold standard (Fig. 2). Owing to the massive amount of air in the whole chest and subcutaneous region, a Fuhrman drain was placed on the midclavicular line, which permitted patient extubation. The young boy was then admitted to PICU for closer monitored observation for 2 days. He was shifted from the PICU to the conventional ward where he stayed for 2 days more. He only complained of subcutaneous emphysema. The drain was removed on day three before joining the pediatric ward.

Throughout the examination, a PEEP was maintained via an APL valve on the KAB™ CO_2_ absorber device. This plastic valve is designed to apply a positive pressure of up to 50cmH_2_O in proportion to its closure. Unfortunately, it is not graduated and, as we shall see later, was in fact much more sensitive than the anesthetist thought.

## Discussion

Pneumothorax is uncommon in the pediatric population without risk factors [1]. They occur mainly due to external causes or underlying lung disease. External causes are mainly iatrogenic and cause by invasive interventions such as central venous catheter insertion, by intubation procedure or inadequate ventilation parameters for example. The underlying conditions are well describe in adult population but remains more unclear in pediatrics. The factors contributing to pneumothorax in the non-intubated neonates and children are rare. in most cases, they are due to idiopathic respiratory distress syndrome or idiopathic blebs. There are 2 types of pneumothoraxes, classified as primary or secondary. Secondary pneumothoraxes are due to underlying pulmonary diseases such as asthma, COPD, cystic fibrosis, interstitial diseases, infections or congenital lung disease. Primary pneumothoraxes result from the absence of evidence implicating the lungs condition after investigation exams. Barotraumatism is a grouping of pulmonary aggressions, of which pneumothorax can be a manifestation. Barotraumatism itself is part of an entity called ventilator-induced lung injuries (VILI). Even if it was not mechanical ventilatory, repeated manual ventilation under positive pressure could lead to similar issues. Pneumothorax is one of the many presentations of VILI. The concept of mechanical ventilation injuries was first described by John Fothergill in 1744; however, in 1988 Dreyfuss reported the influence of high pressure, volumes, and PEEP on ventilated lungs [2]. A few years later, the term VILI appeared with the current definition. This entity is subdivided into the following four mechanisms: barotrauma, volutrauma, atelectrauma, and biotrauma. Studies on these four entities assisted us in the current management of ventilated lungs, particularly in acute respiratory distress syndrome. We will develop more on barotrauma, which played a major role in this case presentation.

To the best of our knowledge, this is the first reported case of complete pneumothorax, pneumomediastinum, and pneumoperitoneum that occurred during a cardiac MRI examination under general anesthesia with assisted ventilation. Although cases of pneumothorax in young children admitted to PICU and under mechanical ventilation were found; however, not with such a presentation. Nonspontaneous pneumothorax incidence may vary from 1 to 2% in the neonatal intensive care unit to 40% in young patients with respiratory distress syndrome [1, 3]. It is commonly due to external or predisposing factors. External causes are mainly traumatic or iatrogenic. The two major causes are the intubation process and central venous access. The other factors are the use of excessive pressure in the respiratory tracts (barotrauma), inadequate respiratory parameters (volotrauma, atelectrauma); and traumatic surgical events near the diaphragm or chest. Predisposing patient factors such as congenital malformations, immature respiratory system (lack of surfactant [biotrauma]), and low birth weight, are related risks to developing pneumothorax [3]. However, perioperative pneumothorax remains uncommon owing to the understanding of the predisposition risks and associated treatments. The use of modern equipment such as sonography for the insertion of central venous access or low-pressure cuffed tracheal tubes in young children contributes to reducing some external risk factors. Medical literature is limited regarding the challenges faced by anesthetists in making the appropriate diagnosis. Clinically-associated signs are poor and unspecific. Clinical presentation depends on the severity of pneumothorax. Associated physical signs are decreased chest extension, diminished breath sounds, enlarged hemithorax on the affected side, and absent tactile or vocal fremitus or subcutaneous emphysema. Use of accessory respiratory muscles; majored ventilatory labor; and hemodynamic compromises (tachycardia, hypotension) are generally severe when pneumothorax occurs during mechanical ventilation. Complementary findings include peripheral low blood saturation or hypoxemia in the arterial blood gasses. These clinical signs are often masked by general anesthesia. For example, it is hard to detect variations in heart rate or respiratory rate under general anesthesia and even more when we use mechanical ventilation. The unbalanced chest movement is a poor and unspecific sign (obstruction, non-effective curarization) and it may be challenging for anesthetists to detect owing to the restricted view of the patient’s body. The gold standard diagnostic aid is radiology and depends on the clinical presentation (stable or unstable patients) and the availability of bedside ultrasonography. If available, echography must be used owing to its better sensitivity and shorter waiting time than radiographs. In unstable patients, the radiologic examination should not delay the emergency treatment that consists of rapid needle thoracentesis.

### Case analysis

As we analyze this case, it appears that multifactorial causes occurred. We will discuss all of the identified causes hereafter.

#### Human factors

Human factors are involved in most errors. Anesthetists who are in charge of the patient in the perioperative period may perform some errors. Keeping in mind that we are human and that humans are responsible for adverse effects. In a review in 2014 on Neonatal anesthesia, Kovatsis reported all the common complications that occurred in children and neonates [4]. He showed that human factors accounted for nearly 40%, with errors in judgment and the failure to check materials as the two most common errors. Nowadays, checklists are progressively implemented and global hospital certifications are spreading widely. All with one main goal, improving communication between healthcare professionals for reducing human factors errors. Auroy discussed the relationship between experiences in pediatric anesthesia and complications [5]. He showed that complications are often due to inadequate experience in anesthetic techniques in children. More experience in pediatric anesthesia leads to fewer complications. It seems to be a number under which you lose your ability skills. This number may vary with the surgery performed and age of the patient. Based on this observation, specific reference centers were opened in some countries [4–6]. In this case, the anesthesia team was uncomfortable by this relatively uncommon procedure. The anesthesia team in charge of the patient consisted of a final-year anesthesia student closely supervised by a board-certified anesthesiologist performing pediatric procedures on a daily basis. The student used the equipment on a weekly basis, as he performed several examinations or procedures outside the operating room. Given the small number of these procedures, few qualified anesthesiologists are familiar with them. Unfortunately, the reference persons were not present on the day of the incident. No one felt the need to postpone this seemingly innocuous examination. The cardiac MRI under general anesthesia required 25 to 30 series of 10-second apneas, and lasts around 30 to 45 min. During these brief apneas, exhaled CO2 increases. Therefore, apneas are followed by periods of rapid manual balloon ventilation to return to physiologic exhaled CO2 thresholds. Since the imposed respiratory rate is higher during these periods, the expiratory time is inevitably shortened. If the exhalation time is too short, air may be trapped and end-tidal pressure may rise, causing pneumothorax.

Excessive ventilatory pressure had probably raised over a critical limit owing to the lack of reliable insufflation pressure monitoring. A well-established concept in pediatric anesthesia is the “educated hand” concept, which was initially published by Egbert and Bisno in 1967 [7]. It argues that experienced anesthetists can detect variation in pulmonary compliance more than inexperienced anesthetists. Nevertheless, this is a constantly debatable argument in the literature since its first publication; however, is based on the same observation that the number of pediatric anesthesia has a major role [8]. The position of the patient in the MRI tube can also cause visibility problems. Indeed, even if the ventilator circuit has normal length tubes (1.8 m), as the patient is located inside the tube, there is not much space to appreciate the patient’s thorax. The desired air/02 and Sevoflurane mix is directly delivered to the KAB™ CO_2_ absorber at 6 L per minute, so we don’t have to use long pipes. This allows the anesthesiologist to manually ventilate the patient while remaining at the patient’s feet for the entire duration of the examination.

#### Environment

If pediatric anesthesia is associated with high risk when there is an inexperienced team that manages the child, the environment has also an important role. It was well described by Kovatis in its review [4]. When young patients have to undergo anesthesia in a procedural area outside the operating room or PICU, risks are greater. The gold standard rule is to maintain the same level of monitoring performed in the operating room. This facilitates the detection of early vital changes. The concept of bringing resources to the patient was the response to minimize risks, especially for neonates and preterm infants. However, resources and equipment such as MRI or scans cannot be displaced. That is why we have to perform anesthesia in remote areas.

#### Materials

**Fig. 3:**
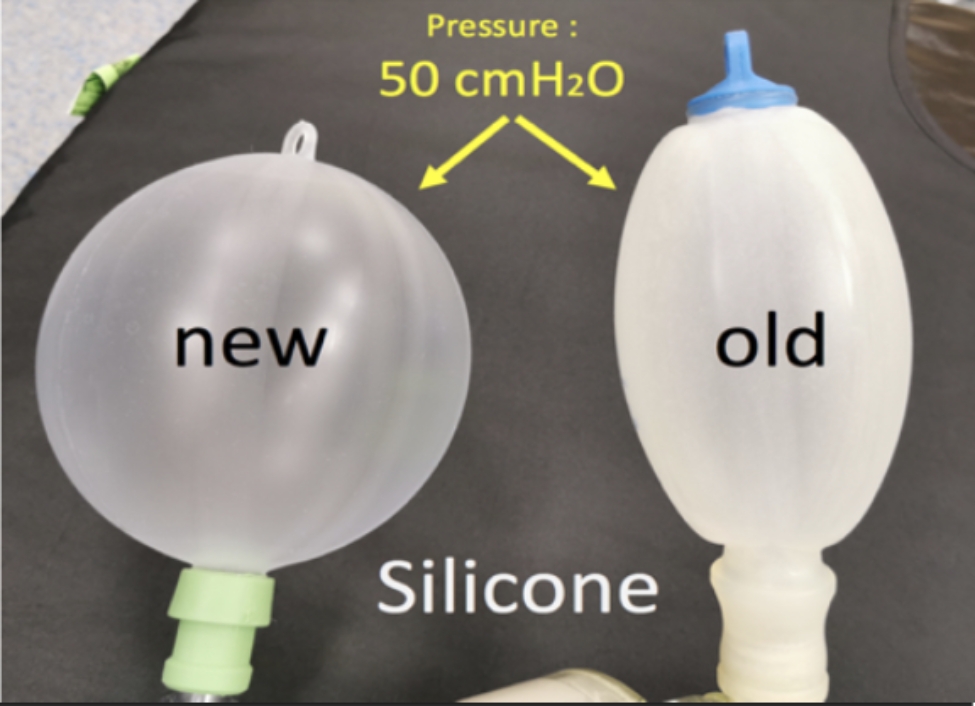
Difference in compliance with the same pressure applied (50 cm of water). The left balloon is a new one and could alert the anesthetist by distending itself. However, the right balloon, the old one, cannot and does not inform of elevated pressure

In the literature, incidents attributed to material issues or defects have been reported. Sabar et al. described a kinked reservoir tube [9], while Smith and McEwan described bilateral tension pneumothorax with a defective bacterial filter [10, 11]. The material was different from the routine material. We normally use respirators with short coaxial breathing pipes, and our monitoring systems are integrated into these respirators. For MRI examinations, we use portable monitors that differ slightly in design and interface, and a modified Mapleson D breathing circuit. All these different devices require time to familiarize ourselves with when they are not used on a routine basis. The Mapleson D-based circuit is connected to a fully plastic IRM-compatible device (KAB™ CO_2_ Absorber) (Fig. 1). This device contains a soda lime container, adjustable pressure-limiting valve, dedicated inspiratory and expiratory valves, and self-inflated balloon. It provides patient transportation or anesthesia gas maintenance under general anesthesia with the advantage of a compact system with good visualization of all components. This device is weekly used for procedures and many years without any reported incident. In this case, it appeared that the ventilation balloon was old and had lost its inherent compliance (Fig. 3).

**Fig. 4:**
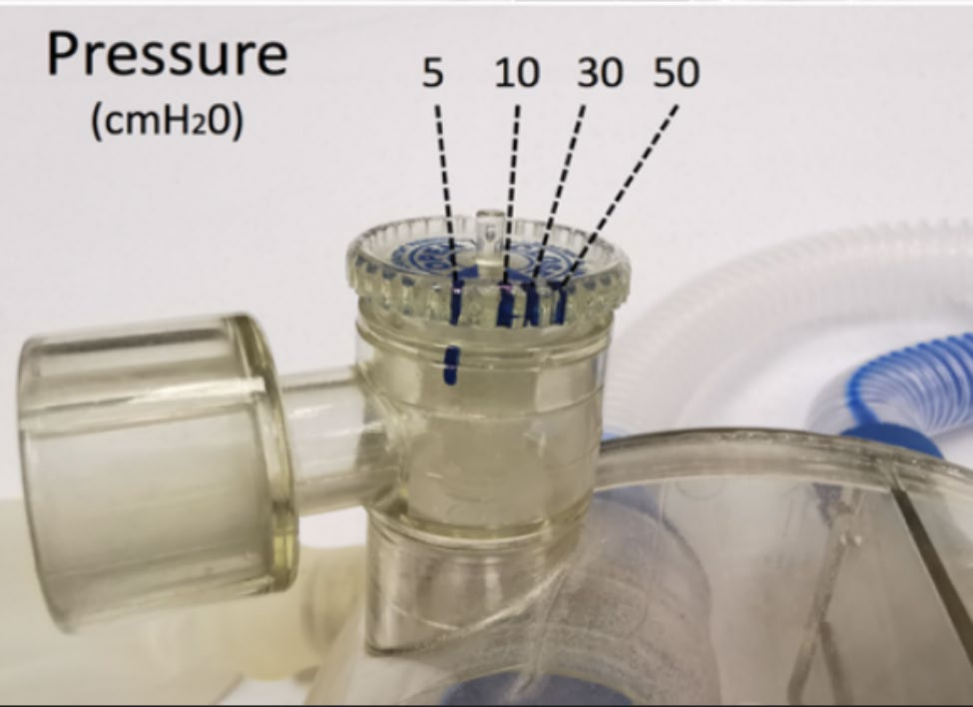
The plastic adjustable pressure-limiting (APL) valve with marks that show the nonlinear rise in pressure within the last 30° of rotation. These marks are made when we connect a manometer and obtrude the expiratory circuit

One of the goals of a compliant balloon is the visual alert of a raise in pressure. In combination with a pressure valve, the balloon is designed for maintaining a constant pressure determined by the valve. When a pressure of 20 cmH_2_O is applied on the pressure valve and the expiratory circuit is obtruded, the balloon inflates while maintaining the maximum expiratory pressure we choose on the valve. The balloon was in poor condition because it was not included in the maintenance list of the operating room materials. All equipment used in non-operating rooms is collected by nurses after use for reconditioning or resterilization. In analyzing this event, we realized that the equipment used for MRI, although considered an off-site workstation, was not part of this maintenance and reconditioning loop. Moreover, the valve used was made of plastic and used in special environments such as MRI. The precision of this valve is not a linear one like the classic APL valve used routinely and it is designed to close in only one turn. There is no graduation on it and the valve is turned 330° to put 5 cmH_2_0 pressure (Fig. 4).

The pressure was then raised to more than 50 cmH_2_O when we complete the turn. We argued that this extreme sensibility of the valve when we pass the 330° turn in addition to the use of a non-compliant balloon and multiple manual ventilation with positive pressure to permit apneas during MRI has, undoubtedly, led to barotraumatic lung injuries. As mentioned in the medical history of the child, there was an ASD, which probably played a protective role for the patient. We hypothesized, with no proof, that it is possible that the maintenance of PEEP and the intermittent use of manual positive pressure ventilation above 50 cm H_2_O could lead to cardiac arrest due to a significant reduction in cardiac output. The use of a high FiO_2_, the presence of an ASD and the diffusion of air into the peritoneum and subcutaneous tissues prevented any repercussions on the right heart and thus avoid this cardiac arrest and severe desaturation from occurring. However, no brain damage owing to the passage of gas bubbles from the right to the left side of the heart has been reported. The orotracheal cuffed tube used had an internal diameter of 4.5 mm and did not permit any gas leak when its position was confirmed after intubation. An uncuffed tube with a bigger internal diameter may have allowed air leaks all around the tracheal tube; therefore, may have protected the boy’s lungs. A few weeks after this incident our service bought new monitoring (ECG, NIBP, SpO_2_) with a large repeater screen to allow us to better manage patients. Moreover, our institution has also bought a fully MRI-compatible respiratory device for the next procedure. The cost of the anesthetic device is approximately 100 000 dollars and shall be used by both the PICU and anesthesiology services.

After a step-by-step analysis of this case report, it seems more appropriate to avoid anesthesia as much as possible in these frail patients. Performing the examination without multiple apnea and thus without anesthesia would be the solution. Therefore we would limit the complications associated with airway management, the need for monitoring equipment or ventilators, and the influence of the human factor. Several teams of radiologists have shown that apnea is not always essential to obtain images allowing a correct diagnosis in the follow-up of many congenital heart diseases in children [12, 13]. Images can be obtained in real time while the patient remains breathing. The examination time is thus greatly reduced, with the double advantage of less disturbing the radiological program and, above all, of avoiding anesthesia by relying on the patient’s cooperation. Sedation may still be necessary in certain conditions, especially in young children. In these cases, the supraglotic device may be a less invasive alternative to intubation and should be considered in conjunction with real-time MRI.

## Conclusions

Through this case, the importance of a safe and well-known environment for anesthesia is highlighted. Certainly, the human factor is the major risk factor for patient safety. Such incidents are uncommon and quite rare in medical literature owing to underreporting. We developed checklists, formations, and alarms to mitigate risks. In some hospitals and countries, the practice of bringing resources to patients has gained favor and shown no difference in mortality [4]. For example, some interventions are performed directly in PICU to prevent inherent risks of patient transportation. The real impacts of such progress are hard to analyze owing to underreporting.

The clinical signs of pneumothorax are well-established; however, general anesthesia could mask most of them. Routine monitors can significantly assist with alarms and visual trends; however, have some limits. In our case, airway pressure monitoring was not available with the KAB™ CO_2_ absorber and thus no alarm could warn us. The capacity skill to detect a variation of pressure in the balloon (the educated hand) is hard to master and is debatable [7]. The number of pediatric anesthesia and experience are also important to prevent incidents. With more experience in pediatric anesthesia, awareness and promptness to detect any abnormalities increase [5]. In light of the above, some substantial changes have been made to avoid such situations and provide more safety for young patients. New MRI monitors were bought, and balloons were changed to include in the classical maintenance circuit as a part of the equipment of the operating room. Moreover, a presentation was prepared for all the persons in charge of such procedures to avoid misinterpretation of equipment. Finally, a dedicated specific MRI ventilatory machine was ordered and made available. Indeed, the cost of the equipment needed to care for these fragile patients should never be at the expense of safety. All of these measures guarantee maximal safety for the staff and patients.

We are all conscious that mistakes are frequent and are part of human beings so we put some alarms and checklists to avoid or correct them. This article aims to learn from mistakes, not relying on our acquired skills, and perpetually keep advancing through education.

With today’s imaging techniques, the need for general anesthesia or deep sedation must be reconsidered to assess the risk-benefit ratio. Eliminating the need for systematic anesthesia would reduce the number of incidents and the resources required for imaging. This would be a significant benefit to patient safety.

## Data Availability

not applicable.

## Abbreviations

MRI: Magnetic resonance imaging
FIO_2_: Fractional inspired oxygen
APL: Adjustable pressure-limiting
SPO_2_: Saturation and pulse oximetry
ECG: Electrocardiogram
NIBP: Non-invasive blood pressure monitoring
EtCO2: End tidal CO2
PICU: Pediatric intensive care unit
PEEP: Positive end expiratory pressure
ASD: Atrial septal defect
VILI: Ventilator-Induced lung injuries
ARDS: Acute respiratory distress syndrome
